# Anaerobic Capacityestimated in A Single Supramaximal Test in Cycling: Validity and Reliability Analysis

**DOI:** 10.1038/srep42485

**Published:** 2017-02-13

**Authors:** Willian Eiji Miyagi, Rodrigo de Araujo Bonetti de Poli, Marcelo Papoti, Romulo Bertuzzi, Alessandro Moura Zagatto

**Affiliations:** 1 Laboratory of Physiology and Sport Performance (LAFIDE), São Paulo State University (Unesp), School of Sciences, Department of Physical Education, Bauru – SP, 17033-360, Brazil; 2 Post-Graduate Program in Movement Sciences, São Paulo State University (Unesp), Institute of Biosciences, Rio Claro – SP, Brazil; 3 School of Physical Education and Sport of Ribeirão Preto (EFEERP), University of São Paulo (USP), 14049907 – Ribeirão Preto, SP, Brazil; 4 Endurance Sports Research Group (GEDAE), School of Physical Education and Sport, University of São Paulo (USP), 05508030, São Paulo - SP, Brazil

## Abstract

The aim was to verify the validity (i.e., study A) and reliability (i.e., study B) of the alternative maximal accumulated oxygen deficit determined using onlya supramaximal effort (MAOD_ALT_)to estimate anaerobic capacity [i.e., estimated by the gold standard maximal accumulated oxygen deficit method (MAOD)] during cycling. In study A, the effects of supramaximal intensities on MAOD_ALT_ and the comparison with the MAOD were investigated in fourteen active subjects (26 ± 6 years). In study B, the test-retest reliability was investigated, where fourteen male amateur cyclists (29 ± 5 years) performed the MAOD_ALT_ twice at 115% of the intensity associated to maximal oxygen uptake (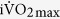). MAOD_ALT_ determined at 130 and 150% of 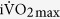 was lower than MAOD (p ≤ 0.048), but no differences between MAOD_ALT_ determined at 100, 105, 110, 115, 120 and 140% of 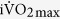 (3.58 ± 0.53L; 3.58 ± 0.59L; 3.53 ± 0.52L; 3.48 ± 0.72L; 3.52 ± 0.61L and 3.46 ± 0.69L, respectively) with MAOD (3.99 ± 0.64L). The MAOD_ALT_ determined from the intensities between 110 and 120% of 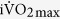 presented the better agreement and concordance with MAOD. In the test-retest, the MAOD_ALT_ was not different (p > 0.05), showed high reproducibility when expressed in absolute values (ICC = 0.96, p < 0.01), and a good level of agreement in the Bland-Altman plot analysis (mean differences ± CI95%:−0.16 ± 0.53L). Thus, the MAOD_ALT_ seems to be valid and reliable to assess anaerobic capacity in cycling.

High-intensity efforts require a high demand of energy, mainly supplied by the non-oxidative metabolic processes^1^. Thus, anaerobic capacity, which is regarded as the maximal amount of energy (i.e., ATP) that can be resynthesized through the phosphagen and glycolytic metabolic pathways^2^, has been considered an important physiological performance determinant in these efforts. Anaerobic capacity evaluation is complex due to the lack of an existing method universally accepted as the gold standard; nevertheless the maximal accumulated oxygen deficit protocol (MAOD) has been considered the most accepted procedure to estimate this parameter^2^. However, despite the scientific acceptance of MAOD to estimate anaerobic capacity^2^,^3^,^4^, the practical application of this method is frequently infeasible due to the large expenditure of time required for its determination.

In 2010, Bertuzzi *et al*.^5^ proposed assessing the MAOD using only a supramaximal exercise test (MAOD_ALT_) in cycling at 110% of peak intensity measured in the graded exercise test (GXT). They assumed the MAOD_ALT_ as the sum of the oxygen equivalents from the glycolytic (i.e., accumulated lactate during the effort) and phosphagen metabolisms (i.e., fast component of excess post-oxygen consumption - EPOC_FAST_)^1^,^6^,^7^. These authors reported that the MAOD_ALT_ was not different from and significantly correlated with conventional MAOD (r = 0.78). In addition, besides the lower time required, the other advantage of MAOD_ALT_ is the possibility of estimating singly the energy contribution engaged in non-mitochondrial pathways.

In order to consolidate the MAOD_ALT_ as an anaerobic capacity predictor, Zagatto *et al*.^8^ investigated its validity and reliability in running. The main arguments given for these authors to investigate the issues were the possible alterations in accumulated blood lactate concentrations ([La^−^]) and EPOC_FAST_ due to different time-to-exhaustion at supramaximal intensities (MAOD_ALT_ estimated at 100, 105, 110, 115, 120, 130, 140, and 150% of intensity associated with the maximal oxygen uptake [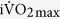]) and to compare the MAOD_ALT_ protocol with the MAOD determined by a gold standard method^2^.The authors found that the MAOD_ALT_ is a valid protocol to estimate anaerobic capacity and it is not altered by the supramaximal effort intensity. Furthermore, it was found that the ideal intensity to determine MAOD_ALT_ was the velocity of 115% of 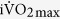 (time-to-exhaustion 156.8 ± 7.6s), which presented a high level of agreement and concordance with the conventional method (r = 0.73) as well as being reliable (i.e., test and retest intraclass correlation of 0.87)^8^.

On the other hand, there is a lack of studies investigating these issues with respect to MAOD_ALT_ in cycling^5^,^9^,^10^, since Bertuzzi *et al*.^5^ only investigated 110% in cycling while Zagatto *et al*.^8^ looked at a range of supramaximal efforts but during treadmill running. This is an intriguing issue because different physiological responses can be observed in cycling compared to running^11^ and MAOD is affected by the exercise mode^12^. It is important to note that one of the main arguments that reinforce the validity of MAOD as a method for assessing anaerobic capacity is that the values remain constant when determined in supramaximal efforts lasting for 2 minutes^3^. In situations of short time-to-exhaustion, MAOD values can be underestimated due to not reaching maximal capacity and an ideal time of between 2–3 minutes has been suggested to exhaust all capacity^3^. However, it has been shown that all-out efforts lasting ~60 s can be used to determine MAOD^13^, stressing the importance of investigating the supramaximal effort duration to estimate anaerobic capacity. Although Zagatto *et al*.^8^ have already investigated these issues concerning the MAOD_ALT_ in running, it is necessary to know the possible effects in cycling as this ergometer is widely used in anaerobic evaluation. In addition, the importance of anaerobic capacity values expressed relative to active muscle mass has been documented^14^, however for MAOD_ALT_ this has not been reported.

Therefore, the main purpose of the present study was to verify the validity and reliability of the MAOD_ALT_ in cycling. For this purpose, the following were investigated: 1) the effects of supramaximal intensities on MAOD_ALT_ and the comparison with conventional MAOD; 2) the test-retestreliability. In accordance with the running results^8^ it was hypothesized that the MAOD_ALT_ would not present differences from MAOD, would be independent of the supramaximal effort, and would be reproducible.

## Results

All subjects achieved at least two criteria to confirm maximal oxygen uptake (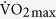) determination^15^ (Table 1).

### Study A

The variables obtained in the supramaximal efforts are presented in Table 2. No significant differences were found in the parameters that involved the glycolytic [i.e., blood lactate concentration and oxygen equivalents of the glycolytic metabolic pathway (E_[La]_)] or phosphagen [i.e., τ_1_, amplitude and oxygen equivalents of the phosphagen metabolic pathway (E_PCr_)] metabolic pathways. Significant differences were found only for exercise intensity and time-to-exhaustion in the supramaximal efforts.

**Figure 1:**
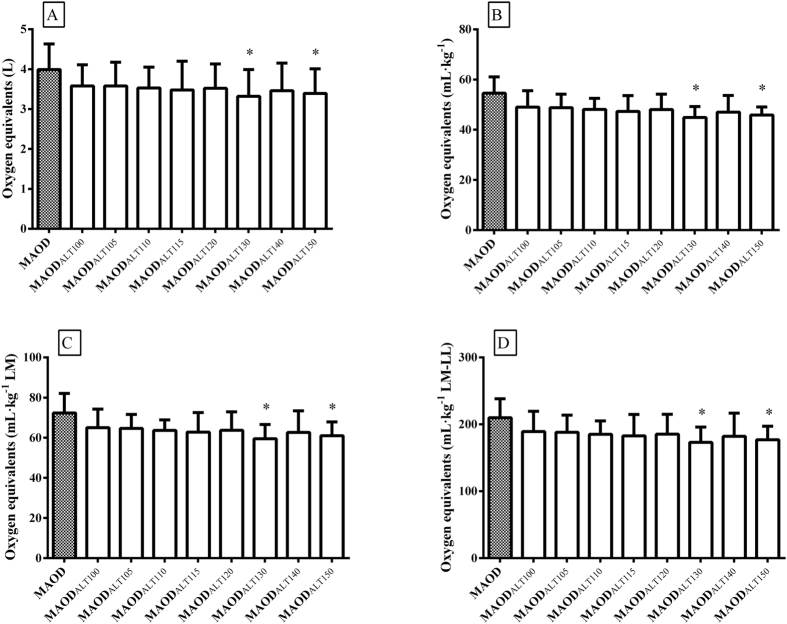
Comparison of the maximal accumulated oxygen deficit determined by the conventional method (MAOD) and alternative method (MAOD_ALT_). The MAOD_ALT_ was determined at intensities of 100, 105, 110, 115, 120, 130, 140, and 150% of the intensity associated with maximal oxygen uptake (MAOD_ALT100_, MAOD_ALT105_, MAOD_ALT110_, MAOD_ALT115_, MAOD_ALT120_, MAOD_ALT130_, MAOD_ALT140,_ and MAOD_ALT150_). The MAOD and MAOD_ALT_ values are presented in absolute (**A**), relative to body mass (**B**), lean mass (LM) (**C**), and lean mass of lower limb values (LM-LL) (**D**). *p < 0.05 to MAOD.

Figure 1 shows the values of MAOD and MAOD_ALT_ presented as absolute, relative to body mass, lean mass, and lean mass of lower limbs. In general the values of MAOD_ALT_ were similar to conventional MAOD, but significant differences were found only for determination using the intensities corresponding to 130 and 150% of 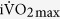 (p ≤ 0.048). In addition, all MAOD_ALT_ values presented moderate and significant correlations with MAOD when expressed in absolute values (r = 0.54 to 0.68; p < 0.05); the MAOD_ALT115_ showed the highest correlation coefficient (r = 0.68; p < 0.01) (Table 3). When expressed in relative lean mass of lower limb values, the MAOD and MAOD_ALT_ only showed moderate and significant coefficients of correlation for MAOD_ALT100_, MAOD_ALT115,_ and MAOD_ALT140_ (r = 0.56 to 0.62; p < 0.05). However, significant correlations were not found between the MAOD_ALT_ and MAOD when expressed relative to body mass and lean mass (Table 3). In addition, considering the comparisons and agreement analysis (Fig. 1 and Table 3), the MAOD_ALT_ determined from the intensities of 110 to 120% of 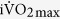 showed the best results. However, only the intensity of 115% of 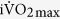 was used to determine the MAOD_ALT_ in Study B (Table 3) to test the reliability based on the highest coefficient of correlation with the “gold standard” MAOD technique (Table 3).

### Study B

**Figure 2:**
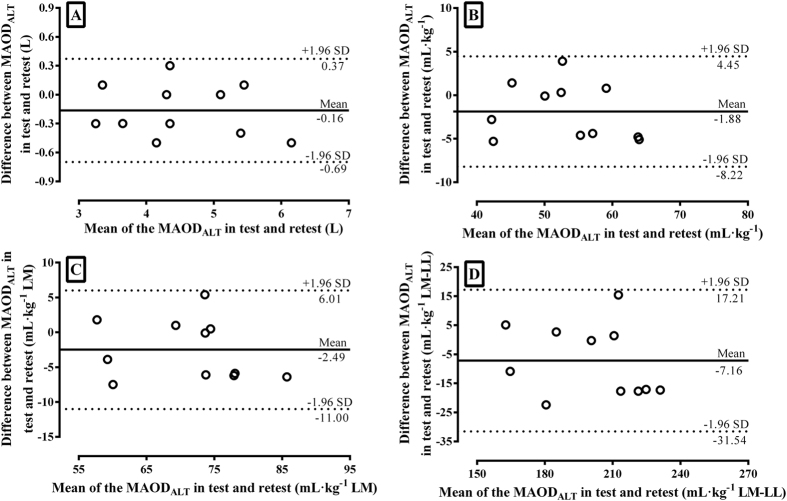
Bland-Altman plot analysis with MAOD_ALT_ determined in test and retest. The MAOD_ALT_ values expressed in absolute (**A**), relative to body mass (**B**), lean mass (**C**), and lean mass of lower limb values (**D**).

The 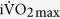 was 280.1 ± 40.5 W (CI95% = 252.9 to 307.3 W). Table 4 shows the MAOD_ALT_ values determined in the test and retest. No significant differences were found in MAOD_ALT_ expressed in absolute or relative values (p > 0.05) (Table 4). In addition, significant correlations and a good level of agreement were found, evidenced by the mean of differences near to zero (Fig. 2). The E_[La]_ and E_PCr_ were not different and also demonstrated significant correlations (Table 4).

## Discussion

The main finding of the study was that MAOD_ALT_ was in general similar and moderately and significantly correlated with the MAOD. Despite the exercise at 130 and 150% of 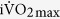 leading to lower MAOD_ALT_ values, its determination was not altered for 100, 105, 110, 120% and 140% of 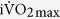 when expressed in absolute values. However, similar to that found in running^8^, the exercise intensities at 110–120% of 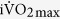 were considered the ideal to determine MAOD_ALT_ in cycling, presenting concordance with MAOD. In addition, the MAOD_ALT_ estimated at 115% of 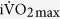 presented high test-retest reliability, reinforcing that the MAOD_ALT_ is a valid procedure to estimate anaerobic capacity in cycling using only one supramaximal effort.

The study by Bertuzzi *et al*.^5^ was the first to compare the MAOD and MAOD_ALT_ in nine healthy male subjects, reporting the validity of MAOD_ALT_ to assess anaerobic capacity in cycling. These authors used six submaximal efforts to determine the MAOD, whilst using at least ten submaximal efforts has been suggested^2^. In the present study, the MAOD was determined using ten submaximal efforts to construct the linear regression from the 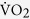-intensity relationship^2^, which reduces the random errors and increases the confidence in the determination of MAOD^2^,^3^. Thus, the results showed that the MAOD_ALT_ remains appropriate to estimate the MAOD, which is considered the most accepted technique to estimate the anaerobic capacity. Despite the relevant findings presented by Bertuzzi *et al*.^5^, these authors determined the MAOD_ALT_ only from the intensity of 110% of peak intensity measured in GXT. Therefore, the use of different exercise intensities and the reliability in the test-retest analysis performed in the current study were used to solve some of these matters relevant to the practical use of the procedure, but under-investigated until the present date in cycling.

The lower values of MAOD_ALT_ estimated at 130 and 150% of 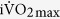 are similar to those reported by Medbø *et al*.^3^, who verified that when the supramaximal effort is at least two minutes long the MAOD values remain constant, emphasizing the anaerobic capacity concept. The lower MAOD_ALT_ values observed at supramaximal efforts with short duration could be related to the maximal ATP resynthesis impaired by the glycolytic pathway, due to several factors related to fatigue in high-intensity efforts, such as acidosis^3^. Thus, the significant differences observed at intensities above 120% of 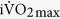 (i.e., MAOD_ALT130_ and MAOD_ALT150_) in the present study could be related to these processes involved in the fatigue at intensities performed until exhaustion with short duration (i.e., less than two minutes). Possibly, this did not allow the maximal depletion of energetic substrate stores engaged in the glycolytic and phosphagen metabolic pathways. In addition, the power-duration relationship might be affected by the population, It is relevant to consider that as the MAOD_ALT_ between 100 and 120% of 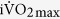 did not differ and were moderate and significantly correlated with MAOD, the time-to-exhaustion range varying around 164s (i.e., 2.74 ± 0.56 min for 120% of 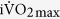) to 313s (i.e., 5.22 ± 0.99 min for 100% of 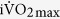) could be used to determine the MAOD_ALT_ in cycling. Despite the fact that MAOD_ALT_ estimated at 140% of 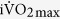 did not differ from MAOD, this exercise intensity must be avoided, as the time-to-exhaustion varies around 110s (i.e., 1.83 ± 0.26 min) and impairs anaerobic capacity estimation due to acidosis-induced fatigue.

The issue of the normalization of anaerobic capacity values for muscle mass involved in the exercise appears to be an important factor to be considered. For example, Hill and Vilgren^16^ showed that the MAOD is sensitive to muscle mass involved in exercise, when comparing the values obtained in running and cycling^16^. In this sense, some studies have investigated the use of MAOD values expressed relative to active muscle mass involved in cycling^17^,^18^. In the present study, moderate (i.e., coefficient of correlations between 0.54 and 0.68) and significant correlations were observed in MAOD_ALT_ values expressed in absolute units and relative to lean mass of the lower limbs (LM-LL) (Table 3), but not when normalized by the total body mass or lean mass (LM). A possible explanation could be related to the differences between the two methods of determination of the anaerobic metabolism. The MAOD_ALT_ is based on the sum of the oxygen equivalents corresponding to glycolytic and phosphagen pathways estimated during the supramaximal effort^5^,^8^,^19^,^20^. The latter takes into account an energetic equivalent expressed relative to the individual’s body mass (i.e. mL·kg^−1^ of body mass). Therefore, the body mass of the individual will only be considered in the calculation when these values of oxygen equivalents are expressed in absolute units. On the other hand, values related to body mass could also influence the results because they include values of fat and bone mass, decreasing MAOD_ALT_ values of individuals with higher fat percentage. Likewise, results may be influenced when MAOD_ALT_ values are expressed relative to total lean mass because they include both active and inactive muscles. We observed that levels of association increased when MAOD values were expressed relative to LM and LM-LL, and statistical significance was only observed for the latter (Table 3). Even with moderate correlations observed, it is possible to suggest that this result perhaps provides more evidence indicating the validity of the protocol because it takes into account active muscle mass during exercise. Although moderately active individuals participated in the present study, another explanation for the results found in relation to normalization of MAOD and MAOD_ALT_ values could be related to possible differences in metabolic adaptations resulting from training. Pizza *et al*.^17^ found that subjects undergoing resistance training presented higher MAOD values relative to lean mass of lower limbs than those trained in endurance and untrained individuals. These authors suggested that metabolic adaptations such as increased amounts of glycolytic and phosphagen enzymes may contribute to higher MAOD values, in addition to active muscle mass.

The main findings of study B were that MAOD_ALT_ determined from 115% of 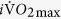 showed no significant differences in the test and retest (effect size between −0.06 and 0.24; coefficient of variation between 4.1 and 4.5%), high reproducibility (i.e., intraclass correlation coefficient between 0.81 and 0.96), and a good level of agreement (mean differences ± CI95%:−0.16 ± 0.53 L). The MAOD is an evaluation protocol used to estimate the anaerobic capacity that demonstrates reproducibility^4^. Weber and Schneider^4^ investigated the reproducibility of the MAOD determined from the intensities of 110 and 120% of 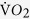 peak and found high reproducibility (i.e., interclass correlation coefficient of 0.95 and 0.97, respectively). In the same way, our findings for MAOD_ALT_ are similar to those for running^8^, which showed that the MAOD_ALT_ presented a mean difference very close to zero in the Bland-Altman analysis (Fig. 2), a small effect size (<0.2)^21^, and high intraclass correlation coefficient^22^, attesting that the MAOD_ALT_ seems to be reliable and reproducible (Table 4). However, to our knowledge, this is the first study to investigate and demonstrate the reproducibility of MAOD_ALT_ and the respective contributions of the glycolytic and phosphagen metabolisms in cycling.

Quantification of anaerobic capacity through the MAOD method should be reliable enough to allow evaluation and monitoring of possible changes related to training^4^. The MAOD_ALT_ seems to be an advantageous tool for use during routine training because it allows the estimation of anaerobic capacity in only one supramaximal exercise session. Furthermore, the MAOD_ALT_ allows discrimination of the glycolytic and phosphagen metabolisms, enabling analysis of the specific responses in each metabolism^23^ and it can be considered a sensitive enough procedure to distinguish the “anaerobic” capacity in individuals with different training levels^19^. Regarding the reliability, although the points obtained from the differences between MAOD_ALT_ values are distributed around the systematic error, there was a limit of agreement in test-retest corresponding to ±0.54L (Fig. 2). These values corresponded to ~10% of the mean values obtained for the MAOD_ALT_ in study B. Thus, one should use caution when the MAOD_ALT_ value is used, for example, to verify possible responses to training, especially if the pre- and post-training differences are less than these values. However, It has been shown that anaerobic capacity can be improved by 16^24^ and 28%^25^ with six weeks appropriate training in moderately active men. Nevertheless, it is important to note that the limits of agreement from the absolute and relative MAOD_ALT_ values in the present study were lower than observed for the conventional MAOD in the test and retest condition^26^ and when the MAOD_ALT_ is used to estimate the conventional MAOD^5^. Nevertheless, another measure of reliability was also favorable, showing a low coefficient of variation of ~4%, which is lower than the findings for the conventional MAOD^26^,^27^.

A possible limitation of the study was that although the MAOD_ALT_ values obtained at other supramaximal intensities presented significant associations with MAOD, the reliability was only tested at 115% of 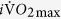. Furthermore, the glycolytic pathway is based on blood lactate concentrations, which do not represent the exact stoichiometry between lactate formation and ATP resynthesis^9^. As an alternative method, intramuscular metabolites could be used to quantify the anaerobic contribution during the exercise. The muscle tissue used for these measurements is usually obtained from small muscle mass. Consequently, it would be necessary to determine the muscle mass that is involved in a specific exercise, which could result in an inaccurate representation of anaerobic metabolism activation^28^. In addition, the muscle biopsy technique requires specialized personnel, and athletes are often uncomfortable participating in studies that use this procedure, especially when many biopsies are required.

Based on the findings of the current study it is possible to reinforce that the MAOD_ALT_ is a valid procedure to estimate the MAOD (i.e. anaerobic capacity) in cycling using only one supramaximal effort. The exercise intensities from 110 to 120% of 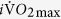 provide the better results of the estimated MAOD by the MAOD_ALT_, mainly when values are expressed in absolute values. Further studies are encouraged to confirm the reliability of MAOD_ALT_ determination using other exercise intensities that are also associated with conventional MAOD.

## Material and Methods

### Subjects

In study A, eighteen moderately active men volunteered to participate. They reported practicing different physical activities such as resistance training, running, cycling, futsal, soccer, Brazilian jiu-jitsu, and swimming and they were not included in any systematic training. The subjects who presented repeated absences (3 subjects) or injuries (1 subject) were excluded. Thus, fourteen individuals completed study A. In study B, eleven mountain-bike cyclists, recreationally trained, volunteered to participate in the study. They reported at least six months experience with a training volume of 121.5 ± 48.5 km·week^−1^ and training sessions three times per week. Table 5 presents the characteristics of the subjects of studies A and B. We used a sample of moderately active men in study A instead of trained individuals due to the difficulty in scheduling several visits of cyclists (i.e., around 10 visits in Study A) to the laboratory to perform the exercise bouts.

All subjects were instructed to avoid alcohol and caffeine ingestion and not to carry out exhaustive exercises for at least 24 h before each session.

We conducted the experiment according to the current International laws. The study was approved by the Ethics Committee of the Sao Paulo State University (Protocol 645.784/2014) and the study was conducted according to the Declaration of Helsinki. Informed consent was obtained from all participants.

#### Experimental design

All tests were performed on a cycle ergometer (Lode-Excalibur Sport, Lode BV, Groningen, The Netherlands) with free choice of cadence between 70–90 rpm, defined in a previous familiarization, as well as saddle settings, and crank distance from handlebars. Subjects were instructed to maintain the preferred cadence with a maximum variation of ±5 rpm in all evaluations. The procedures in each study were applied in an environment with controlled temperature and humidity (20 ± 1 °C and 61 ± 8%, respectively).

In both studies, the subjects were initially submitted to body composition evaluation and a graded exercise test (GXT) to determine the maximal oxygen uptake (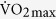) and the associated intensity (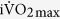).Then, in study A the individuals performed ten submaximal efforts (30–80% of 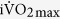) and eight supramaximal efforts (100–150% of 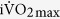). Each session was composed of one submaximal effort, which was used as a warm-up, and one supramaximal effort performed until exhaustion. Thus, eight MAOD_ALT_ values were determined from the supramaximal efforts.

In study B, after the GXT, the individuals performed two supramaximal efforts at the intensity that presented the greatest level of agreement between MAOD and MAOD_ALT_ determined in study A. The interval between each maximal test in both studies was 48 h.

Prior to the beginning of each exhaustive exercise session a POMS questionnaire was applied (i.e., Profile of Mood State)^29^ to evaluate the mood state in order to ensure the best performance in efforts until exhaustion.

#### Physiological measurements

The oxygen uptake (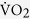) was measured breath-by-breath during all effort tests by a stationary gas analyzer, Quark PFT (COSMED, Rome, Italy). The gas analyzer was calibrated immediately before each test with known samples (5.0% CO_2_and 16.0% O_2_, White Martins^®^, Osasco, Brazil) and room air, while the turbine was calibrated through a three-liter syringe (Hans-Rudolf, USA) following the manufacturer’s recommendations. The 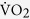 values were smoothed each 5 points and interpolated each 1 second through OriginPro 9.0 software (OriginLab Corporation, Microcal, Massachusetts, USA)^30^. The heart rate (HR) was measured by a transmitter belt coupled to the gas analyzer (Wireless HR 138 Monitor, COSMED, Roma, Italy) and the rate of perceived exertion (RPE) using the 6–20 Borg scale^31^. Blood lactate ([La^−^]) was measured by blood samples (25 μL) collected from the ear lobe at rest and 3, 5, and 7 minutes after each maximal test to determine the peak lactate concentration ([La^−^]_Peak_). The blood samples were collected and stored in *Eppendorf* tubes containing 50 μL of 1% sodium fluoride, stored in a freezer at −20 °C and then analyzed in an electrochemical lactometer YSI 2300 STAT (*Yellow Spring Instruments*, Ohio, USA). In addition, before each session, the subjects remained seated for ten minutes to determine the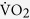 and blood lactate at rest ([La^−^]_Rest_). The resting 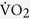 was considered the mean of the final two minutes.

### Body composition analysis

Body composition was measured before all procedures by dual-energy X-ray absorptiometry (DXA) using the Discovery corporal scanner (Hologic, Sunnyvale, USA). The subjects were scanned and the analysis followed the manufacturer’s recommendations. The body segmentation analysis was carried out with the horizontal line positioned above the bowl slightly above iliac crest^32^. The angular lines that define the pelvic triangle were sectioned at the femur, and the vertical line positioned between the legs dividing the two feet. The lean mass of the lower limbs (LM-LL) was considered the sum of the right and left legs, not considering the bone mass values^14^.

## Study A procedures

### Effect of supramaximal effort intensity on MAOD_ALT_

#### Graded exercise test (GXT)

The GXT were carried out to reach exhaustion in ~8–12 minutes^15^. The initial intensity was 75–100 W, with increments of 25 W each 2 minute stage until voluntary exhaustion or the inability to maintain the pre-set cadence^33^. In each exercise stage, the 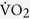 measured during the final 30 s of the stage was averaged. Therefore, the highest average of 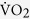 obtained during the test was considered as 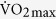, following the criteria to confirm 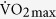 determination^15^. The intensity associated with 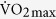 (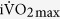) was assumed as the lowest intensity where the 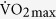 was reached^34^.

#### Submaximal and supramaximal efforts

The subjects performed ten submaximal efforts lasting ten minutes on different days (30, 35, 40, 45, 50, 55, 60, 65, 70 and 80% of 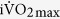) to construct the linear regression from the 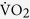-intensity relationship (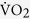). For the higher exercise intensities (i.e., 70 and 80% of 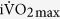), only the effort where a 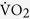 steady state was observed was considered to avoid considering points of the 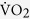-intensity relationship above the anaerobic threshold. The linear regression allowed the estimative of the supramaximal intensity demand and subsequently MAOD determination^2^. The lowest submaximal effort was carried out together with the highest supramaximal effort. Consequently, the second lowest intensity submaximal exercise was combined with the second highest intensity supramaximal exercise and so on. A 10 minutes rest interval was allowed between the submaximal and supramaximal efforts in each session. The submaximal efforts at 70 and 80% of 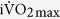 were performed separately so as not to directly affect the supramaximal efforts.

Each subject performed, in different sessions, eight supramaximal efforts until exhaustion at 100, 105, 110, 115, 120, 130, 140 and 150% of 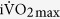.The time to exhaustion was evaluated and the 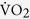 monitored at rest, during the test, and seven minutes after the test, for EPOC_FAST_ analysis. Eight MAOD_ALT_ values were determined, corresponding to each supramaximal effort. The supramaximal intensity at 110% of 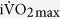 was used to determine the MAOD^4^.

#### MAOD determination

**Figure 3:**
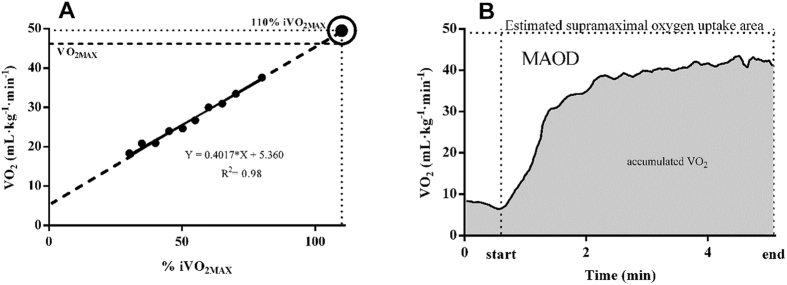
Supramaximal oxygen uptake (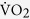) estimated at 110% of the intensity associated with the maximal oxygen uptake (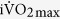) (**A**) and the maximal accumulated oxygen deficit (MAOD) (**B**).

Initially, the linear regression was constructed from the relationship between the submaximal efforts and respective 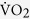. The y-intercept was fixed with the mean of 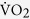 baseline values (rest) (Fig. 3A)^35^. The 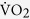 of each exercise intensity was assumed as the 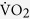 mean of the final two minutes of exercise^2^. To estimate the supramaximal energetic demand, the linear regression was constructed to minimize possible effects related to the number of submaximal efforts, a fixed y-intercept and the non-linearity caused by the slow component of the 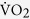 when exercise intensities above the anaerobic threshold were included^2^.

The supramaximal effort of 110% of 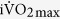 was performed by the subjects and the MAOD corresponded to the difference between the area of the estimated 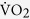 supramaximal (product between the estimated demand and time to exhaustion) and accumulated 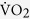 during the test^2^,^3^ (Fig. 3B). The 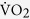 accumulated during the test was calculated using the trapezoidal method. The MAOD values were reduced by10%, corresponding to the body oxygen stores for energy supply^3^,^4^,^5^,^14^.

#### Alternative MAOD (MAOD_ALT_)

The MAOD_ALT_ corresponded to the sum of the oxygen equivalents from the phosphagen (E_PCr_) and glycolytic (E_[La]_) metabolisms^5^,^8^, which were calculated in each session at 100 (MAOD_ALT100_), 105 (MAOD_ALT105_), 110 (MAOD_ALT110_), 115 (MAOD_ALT115_), 120 (MAOD_ALT120_), 130 (MAOD_ALT130_), 140 (MAOD_ALT140_), and 150% (MAOD_ALT150_) of the 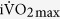. The MAOD_ALT_ values were presented in absolute values, relative to lean mass (LM), and lean mass of the lower limbs (LM-LL).

The E_PCr_ was assumed as the EPOC_FAST_ area, calculated using a bi exponential adjustment (Equation 1), and corresponding to the product between amplitude 1 (A_1_) and time constant1 (τ1) (Equation 2)^1^,^5^,^36^,^37^,^38^,^39^. The E_[La]_was estimated by the difference between the peak and resting blood lactate concentrations, considering the oxygen equivalent of 3 mL·kg^−1^ for each 1.0 mmol·L^−1^ of accumulated lactate above resting level^6^.


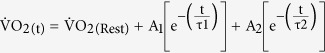



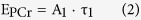


where 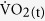 is the oxygen consumption at time *t*, 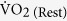 is oxygen consumption at rest, A is the amplitude, and τ is the time constant. 1 and 2 means the fast and slow components, respectively.

### Study B procedures

#### MAOD_ALT_ reliability

In Study B, the subjects performed the body composition evaluation, GXT, and two supramaximal efforts. The supramaximal efforts were carried out at the intensity that presented the best level of agreement between the MAOD and MAOD_ALT_ in study A. All procedures were performed following the same protocol described in Study A, except for the initial intensity in the GXT, which was 100–150 W. The warm-up in this study was standardized at 100 W, lasting five minutes.

### Statistical analysis

The data are presented as mean ± standard deviation (SD) and 95% confidence interval (CI95%). Initially, the data were submitted to the Shapiro-Wilk test to verify normality. In Study A, for comparison of MAOD and the eight MAOD_ALT_ values, Analysis of Variance (ANOVA) One Way for repeated measures was used. The Mauchly’s test was applied to verify the sphericity. In case of sphericity violation, the Greenhouse-Geisser Epsilon correction was used. The ANOVA was completed with the Sidak comparison test, if necessary. In addition, the comparison between MAOD and MAOD_ALT_ was performed with the effect size calculation (*η*^2^), Bland-Altman analysis^40^, Pearson’s correlation test, and typical error. The coefficient of correlation was classified as very weak to negligible (0 to 0.2), weak (0.2 to 0.4), moderate (0.4 to 0.7), strong (0.7 to 0.9), and very strong (0.9 to 1.0)^41^. In study B, for comparison of the values obtained in the test and retest the t test for repeated samples was used. The intraclass correlation coefficient (ICC (3.1); Two-way mixed, consistency, single measures) was used for analysis of reproducibility and Bland-Altman analysis to determine the level of agreement. In addition, the coefficient of variation was calculated by dividing the standard deviation and mean of each participant in the test-retest condition, and then calculating the mean for all subjects. IBM^®^ SPSS 20 software (Chicago, IL, USA) was used for statistical analysis and in all cases a 5% level of significance was considered.

## Additional Information

**How to cite this article**: Miyagi, W. E. *et al*. Anaerobic Capacityestimated in A Single Supramaximal Test in Cycling: Validity and Reliability Analysis. *Sci. Rep.* **7**, 42485; doi: 10.1038/srep42485 (2017).

**Publisher's note:** Springer Nature remains neutral with regard to jurisdictional claims in published maps and institutional affiliations.

## Figures and Tables

**Table 1:** Physiological responses at exhaustion in the graded exercise test.

	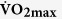 (mL·kg^−1^·min^−1^)	RER	HR_max_ (bpm)	RPE	[La^−^]_Peak_ (mmol·L^−1^)	Total time (min)
Study A	43.4 ± 4.9	1.20 ± 0.06	183 ± 6	18 ± 1	11.1 ± 1.4	12.2 ± 2.5
(n = 14)	(40.5 to 46.2)	(1.17 to 1.23)	(180 to 187)	(17 to 19)	(10.3 to 11.9)	(10.8 to 13.6)
Study B	45.5 ± 7.4	1.21 ± 0.05	188 ± 9	18 ± 2	10.0 ± 2.1	12.8 ± 3.6
(n = 11)	(40.5 to 50.5)	(1.17 to 1.25)	(181 to 194)	(17 to 20)	(8.5 to 11.5)	(10.4 to 15.3)

Values presented as mean ± SD (CI95%). 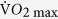 = maximal oxygen uptake. RER = respiratory exchange ratio. HR_max_ = maximal heart rate. RPE = rate of perceived exertion. [La^−^]_Peak_ =^ ^peak lactate.

**Table 2:** Intensity, time to exhaustion, oxygen equivalent of the glycolytic metabolism (E_[La]_), resting lactate ([La^−^]_Rest_), peak lactate ([La^−^]_Peak_), difference between [La^−^]_Rest_ and [La^−^]_Peak_ (Δ[La^−^]), oxygen equivalent of the phosphagen metabolism (E_PCr_), amplitude (A_1_), and time constant (τ_1_)of the bi-exponential adjustment obtained in the supramaximal efforts at 100, 105, 110, 115, 120, 130, 140, and 150% of the intensity associated with maximal oxygen uptake (MAOD_ALT100_, MAOD_ALT105_, MAOD_ALT110_, MAOD_ALT115_, MAOD_ALT120_, MAOD_ALT130_, MAOD_ALT140_, and MAOD_ALT150_).

	100% 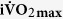	105% 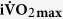	110% 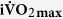	115% 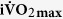	120% 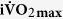	130% 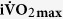	140% 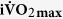	150% 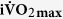	*F*_(7,91)_	p-value
Intensity (W)	225.6 ± 34.1 (206.0 to 245.3)	237.1 ± 35.8^a^ (216.4 to 257.8)	248.5 ± 37.4^ab^ (226.9 to 270.1)	259.6 ± 39.3^abc^ (236.9 to 282.2)	270.8 ± 40.9^abcd^ (247.2 to 294.4)	293.6 ± 44.2^abcde^ (268.1 to 319.2)	315.9 ± 47.7^abcdef^ (288.4 to 343.5)	338.8 ± 51.0^abcdefg^ (309.3 to 368.2)	620.62	0.000
Time to exhaustion (min)	5.22 ± 0.99 (4.65 to 5.79)	4.24 ± 0.26^a^ (3.81 to 4.68)	3.66 ± 0.46^a^ (3.39 to 3.92)	3.15 ± 0.66^ab^ (2.77 to 3.53)	2.74 ± 0.56^abc^ (2.42 to 3.07)	2.09 ± 0.28^abcde^ (1.93 to 2.25)	1.83 ± 0.26^abcde^ (1.68 to 1.98)	1.59 ± 0.22^abcdefg^ (1.46 to 1.72)	109.60	0.000
E_[La]_ (L)	2.30 ± 0.47 (2.03 to 2.57)	2.32 ± 0.52 (2.02 to 2.62)	2.28 ± 0.35 (2.08 to 2.48)	2.22 ± 0.54 (1.91 to 2.53)	2.31 ± 0.46 (2.05 to 2.48)	2.11 ± 0.52 (1.80 to 2.41)	2.21 ± 0.50 (1.92 to 2.50)	2.20 ± 0.46 (1.94 to 2.46)	0.92	0.493
[La^−^]_Rest_ (mmol·L^−1^)	1.17 ± 0.30 (1.00 to 1.35)	1.36 ± 0.41 (1.12 to 1.60)	1.13 ± 0.32 (0.94 to 1.31)	1.25 ± 0.28 (1.09 to 1.41)	1.15 ± 0.27 (1.00 to 1.31)	1.21 ± 0.32 (1.02 to 1.39)	1.26 ± 0.36 (1.06 to 1.47)	1.13 ± 0.17 (1.04 to 1.23)	0.98	0.452
[La^−^]_Peak_ (mmol·L^−1^)	11.71 ± 2.15 (10.47 to 12.95)	11.89 ± 1.72 (10.90 to 12.88)	11.51 ± 1.28 (10.78 to 12.25)	11.33 ± 1.83 (10.27 to 12.39)	11.71 ± 1.78 (10.69 to 12.74)	10.69 ± 1.65 (9.73 to 11.64)	11.30 ± 1.91 (10.19 to 12.40)	11.08 ± 1.28 (10.34 to 11.82)	1.21	0.308
Δ[La^−^] (mmol·L^−1^)	10.54 ± 2.08 (9.33 to 11.74)	10.53 ± 1.75 (9.52 to 11.54)	10.39 ± 1.18 (9.71 to 11.07)	10.08 ± 1.93 (8.97 to 11.19)	10.56 ± 1.92 (9.45 to 11.67)	9.48 ± 1.63 (8.54 to 10.42)	10.03 ± 1.87 (8.95 to 11.11)	9.94 ± 1.27 (9.21 to 10.68)	1.21	0.306
E_PCr_ (L)	1.28 ± 0.20 (1.16 to 1.39)	1.26 ± 0.22 (1.13 to 1.39)	1.24 ± 0.28 (1.08 to 1.41)	1.26 ± 0.29 (1.09 to 1.43)	1.21 ± 0.27 (1.05 to 1.36)	1.21 ± 0.27 (1.06 to 1.37)	1.25 ± 0.28 (1.09 to 1.41)	1.19 ± 0.29 (1.02 to 1.36)	1.06	0.393
A_1_ (mL·kg^−1^·min^−1^)	19.3 ± 3.0 (17.5 to 21.0)	19.3 ± 2.7 (17.7 to 20.8)	18.0 ± 4.1 (15.6 to 20.4)	18.6 ± 3.5 (16.6 to 20.6)	18.3 ± 2.3 (16.9 to 19.6)	17.7 ± 2.4 (16.4 to 19.1)	17.9 ± 2.6 (16.4 to 19.4)	17.4 ± 2.0 (16.3 to 18.6)	1.52	0.225
τ_1_ (min)	0.92 ± 0.11 (0.85 to 0.98)	0.90 ± 0.16 (0.81 to 0.99)	1.01 ± 0.42 (0.77 to 1.26)	0.94 ± 0.19 (0.83 to 1.05)	0.90 ± 0.09 (0.85 to 0.95)	0.93 ± 0.10 (0.87 to 0.99)	0.95 ± 0.12 (0.88 to 1.02)	0.93 ± 0.13 (0.85 to 1.00)	0.53	0.811

Values presented as mean ± SD (IC95%). ^a^p < 0.05 to 100% 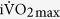. ^b^p < 0.05 to 105% 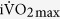. ^c^p < 0.05 to 110% 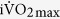. ^d^p < 0.05 to 115% 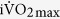. ^e^p < 0.05 to 120% 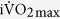. ^f^p < 0.05 to 130% 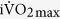. ^g^p < 0.05 to 140% 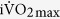.

**Table 3:** Relationship and level of agreement between MAOD and MAOD_ALT_ determined at different supramaximal efforts.

		MAOD_ALT100_	MAOD_ALT105_	MAOD_ALT110_	MAOD_ALT115_	MAOD_ALT120_	MAOD_ALT130_	MAOD_ALT140_	MAOD_ALT150_
MAOD (L)	Coefficient of correlation	0.54*	0.57*	0.66**	0.68**	0.62*	0.55*	0.65*	0.61*
	(CI95%)	(0.01 to 0.83)	(0.06 to 0.85)	(0.20 to 0.88)	(0.24 to 0.89)	(0.14 to 0.87)	(0.03 to 0.84)	(0.18 to 0.88)	(0.12 to 0.86)
	Effect size	0.71	0.67	0.80	0.75	0.75	1.03	0.80	0.96
	Mean of differences (±CI95%)	−0.42 (0.33)	−0.42 (0.33)	−0.47 (0.28)	−0.52 (0.31)	−0.47 (0.32)	−0.68 (0.36)	−0.53 (0.32)	−0.61 (0.32)
	Typical error (L)	0.41	0.40	0.35	0.39	0.39	0.44	0.40	0.39
MAOD (mL·kg^−1^)	Coefficient of correlation	0.44	0.23	0.15	0.27	0.26	−0.20	0.26	−0.08
	(CI95%)	(−0.12 to 0.79)	(−0.34 to 0.68)	(−0.41 to 0.63)	(−0.30 to 0.70)	(−0.31 to 0.70)	(−0.66 to 0.37)	(−0.32 to 0.69)	(−0.58 to 0.47)
	Effect size	0.85	0.97	1.18	1.13	1.03	1.78	1.14	1.78
	Mean of differences (±CI95%)	−5.53 (3.99)	−5.76 (4.30)	−6.47 (4.22)	−7.26 (4.46)	−6.54 (4.44)	−9.69 (4.94)	−7.51 (4.63)	−8.69 (4.33)
	Typical error (mL·kg^−1^)	4.89	5.27	5.17	5.46	5.44	6.05	5.67	5.30
MAOD (mL·kg^−1^ LM)	Coefficient of correlation	0.51	0.31	0.30	0.45	0.43	0.07	0.48	0.33
	(CI95%)	(−0.02 to 0.82)	(−0.26 to 0.72)	(−0.28 to 0.72)	(−0.10 to 0.79)	(−0.13 to 0.78)	(−0.48 to 0.58)	(−0.07 to 0.80)	(−0.25 to 0.73)
	Effect size	0.78	0.93	1.17	0.98	0.92	1.53	0.96	1.37
	Mean of differences (±CI95%)	−7.39 (5.41)	−7.77 (7.08)	−8.72 (5.51)	−9.54 (5.87)	−8.65 (5.85)	−12.82 (6.68)	−9.78 (6.06)	−11.39 (5.71)
	Typical error (mL·kg^−1^ LM)	6.63	7.08	6.75	7.19	7.16	8.18	7.42	7.00
MAOD (mL·kg^−1^ LM-LL)	Coefficient of correlation	0.62*	0.46	0.42	0.55*	0.51	0.18	0.58*	0.34
	(CI95%)	(0.13 to 0.87)	(−0.10 to 0.80)	(−0.14 to 0.78)	(0.04 to 0.84)	(−0.03 to 0.82)	(−0.39 to 0.65)	(0.08 to 0.85)	(−0.23 to 0.74)
	Effect size	0.71	0.81	1.03	0.90	0.85	1.45	0.89	1.36
	Mean of differences (±CI95%)	−20.87 (14.78)	−21.89 (16.30)	−24.90 (15.54)	−27.09 (16.52)	−24.67 (16.56)	−36.95 (19.05)	−27.87 (16.84)	−33.00 (16.56)
	Typical error (mL·kg^−1^ LM-LL)	18.10	19.97	19.03	20.23	20.28	23.33	20.63	20.27

*p < 0.05. ^**^p < 0.01. LM-LL = lean mass of lower limbs.

**Table 4:** Time to exhaustion, alternative maximal accumulated oxygen deficit (MAOD_ALT_), oxygen equivalents from the glycolytic (E_[La]_) and phosphagen metabolisms (E_PCr_) determined in the test and retest condition (n = 14).

	Test	Retest	ES	CV	*p*-value	ICC (CI95%)
Time to exhaustion (s)	182.1 ± 16.2 (171.2 to 193.0)	180.7 ± 25.2 (163.8 to 197.7)	−0.06	4.5%	0.708	0.81^¥^ (0.43 to 0.94)
MAOD_ALT_ (L)	4.42 ± 0.92 (3.80 to 5.03)	4.58 ± 0.96 (3.94 to 5.23)	0.17	4.1%	0.075	0.96^¥^ (0.85 to 0.99)
MAOD_ALT_ (mL·kg^−1^)	52.2 ± 7.5 (47.1 to 57.2)	54.1 ± 8.2 (48.5 to 59.6)	0.24	4.1%	0.082	0.92^¥^ (0.72 to 0.98)
MAOD_ALT_ (mL·kg^−1^ LM)	70.0 ± 8.7 (64.1 to 75.8)	72.5 ± 9.5 (66.1 to 78.8)	0.27	4.1%	0.086	0.89^¥^ (0.63 to 0.97)
MAOD_ALT_ (mL·kg^−1^ LM-LL)	197.1 ± 23.2 (181.5 to 212.7)	204.2 ± 26.2 (186.7 to 221.8)	0.55	4.1%	0.085	0.87^#^ (0.60 to 0.96)
E_[La]_ (L)	2.76 ± 0.56 (2.39 to 3.14)	2.77 ± 0.63 (2.35 to 3.19)	0.01	4.9%	0.896	0.92^¥^ (0.75 to 0.98)
[La^−^]_Rest_ (mmol·L^−1^)	1.1 ± 0.2 (0.9 to 1.2)	1.0 ± 0.3 (0.9 to 1.2)	−0.40	18.5%	0.666	0.16 (−0.46 to 0.67)
[La^−^]_Peak_ (mmol·L^−1^)	12.0 ± 1.7 (10.9 to 13.2)	12.0 ± 2.0 (10.6 to 13.4)	0.00	4.6%	0.882	0.89^¥^ (0.64 to 0.97)
Δ[La^−^] (mmol·L^−1^)	10.9 ± 1.7 (9.8 to 12.1)	11.0 ± 1.9 (9.7 to 12.2)	0.05	5.3%	0.973	0.88^¥^ (0.62 to 0.97)
E_PCr_ (L)	1.64 ± 0.44 (1.34 to 1.93)	1.79 ± 0.51 (1.45 to 2.14)	0.31	12.1%	0.210	0.68^¥^ (0.17 to 0.90)
A_1_ (mL·kg^−1^·min^−1^)	19.4 ± 3.0 (17.3 to 21.4)	19.6 ± 3.6 (17.2 to 22.2)	0.66	5.9%	0.794	0.71^¥^ (0.24 to 0.91)
Time constant (min)	1.00 ± 0.21 (0.87 to 1.14)	1.09 ± 0.20 (0.95 to 1.22)	0.43	14.2%	0.309	0.21 (−0.41 to 0.70)

Values presented as mean ± SD. ^*^p < 0.05 to test condition. ^#^p < 0.05. ^¥^p^ ^< 0.01. [La^−^]_Rest_ = resting lactate. [La^−^]_Peak_ =^ ^peak lactate. (ES = effect size; ICC = intraclass correlation coefficient).

**Table 5:** Characteristics of the participants.

	Age (years)	Height (cm)	Body Mass (kg)	Lean Mass^*^ (kg)	Fat^*^ (%)	LM-LL^*^ (kg)
Study A (n = 14)	26 ± 6	172.4 ± 5.4	73.8 ± 10.8	55.4 ± 6.4	19.4 ± 4.5	19.1 ± 2.4
Study B (n = 11)	28 ± 4	177.0 ± 5.8	85.2 ± 16.1	63.2 ± 11.0	20.5 ± 4.8	22.5 ± 4.4

Values presented as mean ± SD. BM = Body mass. LM = lean mass. LM-LL = lean mass of the lower limbs. 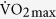
= maximal oxygen uptake. *Estimated by the dual-energy X-ray absorptiometry method.
